# Letter from Our Paris Correspondent

**Published:** 1887-12

**Authors:** 


### FOREIGN CORRESPONDENCE.

FROM OUR OWN CORRESPONDENT.

Paris, France, November 12, 1887.

*Editor Daniel's Texas Medical Journal*:

It might be of interest to the readers of the D. T. M. Journal to hear a few of the comments made by the European medical press upon the subject of the International Medical Congress, recently in session at Washington. The whole meeting seems to be considered among the profession at large as a failure. All the journals make allusions in that direction, while the "Bulletin" and the "Union Medicale" do not hesitate to speak it out in plain language. A general complaint is made about the deplorable dissension among the prominent members of the American profession, and it is freely stated that, with a few exceptions, the leading men of the Congress were only of secondary distinction. It is also generally acknowledged that the American physicians lack that pleasant congeniality so characterisic of the fraternity of the old world. Dr. Hamilton is described as a polite, but rather cold person. In his capacity as general secretary, he is found to be either careless in regard to informing the foreign deputies, or else unprovided with the knowledge of the details almost indispensible to a person of fitness for such a place.

Another matter of special emphasis is the fact that the Congress was made an official meeting by the invitation of foreign representatives as made by the U. S. government. In spite of this official character, the delegates were not received by a reception committee at New York; in spite of this governmental recognition, the U. S. offered none of their public halls for the session, although the legislative bodies were in vacation. On the contrary, the different sections were scattered all over the town, making it almost a matter of impossibility for the foreign congressionists to find them. Besides, the idea of having the general office of the secretary in a common "hotel of travelers," and the meetings in a theatre, seems to be doubly ridiculous to the French minds.

The gala receptions were equally eulogized as a failure, especially for the circumstance that the poor classes of Washington crowded into the halls, carrying off armsful of eatables to swallow them up with best of appetites, while seating themselves in a ring npon the ground. One of the correspondents fears that the Presidential couple, Mr. and Mrs. Cleveland, have their right arms displaced by those 7000 vigorous hand-shakes, etc.

A very ridiculous complaint is made over the circumstance that hardly anybody understood the French, or any other foreign language. It is, however, an exception, a great rarity, to meet a Frenchman speaking anything else but his mother-tongue. If one calls attention to this, the usual reply is about as follows: *Tout le monde sait le Francais, on nous comprend partout.* I suppose they have found out that French does not monopolize America. In respect to the nomination of vice-presidents, everybody found it strange that the majority of elections were made of men who excelled by their absence from the Congress, and who were not men of distinction, even in their own countries.

At the last meeting of the Academy of Sciences Prof. Bouchard gave a detailed account of his researches in the field of practical antiseptics. He said that so far as we have no reliable agents for the disinfection of large absorbing serous cavities, and particularly of the intestinal tracts. Carbolic acid, the sublimate and the biniodide of mercury were excellent and powerful germicides, but their extreme solubility favors absorption before passing through the bowels. On the other hand, their disinfecting qualities are equaled by their tonic influence upon the human body.

If, therefore, we could find some substance of very small solubility, and very little poisonous influence upon the human organism, the question might be considered solid. Prof. B. thinks to have made an important discovery in this direction by experimenting with napthol, (not napthaline). This substance dissolves in a proportion only of 1:500 of water. By adding a little alcohol, the proportion may be increased *ad libitum*. If ingested, a very moderate quantity would suffice to pass through the digestive tube. The question now comes up, how that quantity would influence the tissues? Taking the average adult, he could take 250 grammes before being poisoned. One hundredth ($\frac{1}{100}$) of that weight could, however, suffice to do the work of disinfection. While absolutely speaking, corrosive sublimate exceeds it several hundred times in power, napthol, by means of its enormous non:toxic doses would, in full strength, represent 14-15 times the efficacy of the former. It follows from this, that in all the fermentative affections of the stomache and the bowels, the new drug is certain to play a very important figure, Especially would this apply to the diarrhœal troubles of infancy during the hot months.

Average single-dose in proportion with the body weight 1:2500, single toxic dose 1:1200.

If, according to the professor, a single dose of 2,50 grammes be enough, gr.35-40 ought to do the work in America.

Some very interesting news for the general practitioner comes from the Saint-Indie Hospital, of Bordeaux, and if the efficacy of the treatment proves to be the same in other hands, one of the most rebellious affections would be conquered. The patients have a feeling of heaviness and uneasiness about the stomache, which, during the full process of digestion, develops into a veritable nausea, followed by vomiting. The majority of sufferers vomit every day. The bowels may be regular, but are often inclined to diarrhœal attacks. The stomach, from this excessive fermention, is considerably dilated, and may pass far beyond its normal limits. The gastric trouble may give rise to secondary troubles, among which headaches and the so-called stomachical vertigo are the most frequent and annoying. There is no tenderness over the epigastrium, no vomiting or spitting of blood—in short, no indication of gastric ulcer or cancerous processes. The patient is usually emaciated from the disturbed digestion, and the tongue presents a whitish appearance. A sign of extreme diagnostic value, when present, are little nodosities, which may be found around the articulations of the first and second phalanges of the fingers. Gaseous discharges from the stomach are extremely frequent, and percussion reveals easily the reason for that fact.

Treatment: Exclusive restriction to milk diet, washing out the stomach with weak antiseptic solutions are powerful adjuvants, but the curative means *par excelllence* is the water of Pongues Saint-Leger. It may be given with the drinks of milk, or separately. Its dose is that of the mineral waters generally. By its methodic use some very rebellious cases have yielded within from two weeks to two months' treatment. Usually the vomiting ceases after a few days. As soon as the physician has arrived at that point, he can add eggs to the diet, and, soon after, all food stuffs favoring fecal residue.

In regard to glaucoma, Dr. de Wecker asserts that the term "in flammatory" is to be strictly separated from the affection. True, it may follow an inflammation of the ocular membranes, but it then is only a result. All symptoms are directly referable to the effect of the increased intra-ocular tension. This latter is due to the fact, that the filtering apparatus for the elimination of the used up nutritious fluid is destroyed. This apparatus is situated near the corneal junction with the sclerotic in form of a trabecular tissue allowing the passage of the surplus liquid into the neighboring veins and lymphatics, in about ⅓ of all cases a glacoma commences with certain premonitions. Dr. de Wecker calls this the prodromic form. It is liable to be observed when the subject is yet comparatively young. It may never develop into a full glaucoma, or, at least, very slowly. Operations should, therefore, be postponed. Instillations of eserine (2 per cent sol.), or pilocarpine (5 per cent sol.) by causing a miatic condition and increasing the tension brings about a forced filtration, and hence serves the patient for years. General hygiene is to be strictly enforced. Clinical recognition of that form: tension of affected eye, but little increased, amplitude of accommodation diminished, cornea tarnished on oblique illumination on account of stretching and partial loosening of corneal epithelium. Ophthalmoscopic examination reveals a normal picture, patient complains of an incessant cloudiness, which increases with the intensity of the light. Around a fixed flame he observes a circle of colored bands, like sections of a rainbow. The symptoms disappear at once upon instillation of a myotic, such as pilocarpine. All things causing a dilatation of the pupil lead to an aggravation of suffering.

Dr. Freund, of Strassburg, after a successful trial of over 4000 cases in the hospital, recommends his new manner of manipulation for the completion of the third stage of labor. He says there are two principal modes of treating the matter. The one which interdicts all interference, leaving everything to nature, is usually called the natural method. It gives an average duration of about four hours to the expulsion of the placenta, while Crede's method seeks to accomplish the same thing in one-fourth to one-half hour. Concerning the physiological process of the detachment of the after-birth, we know that it is due to a retro-placental hemorrhage. Crede'a method recommending rubbing, or even kneading of the tired uterus, interferes with the natural and detaching hemorrhage. True, it sets up new contractions, which, in the great majority of cases, succeed to bring about a more speedy delivery. However, during its efforts the womb only imperfectly loosens the secundines leaving numerous pieces behind. It is a statistical fact that the membranes are always less than after the natural process. This will easily show us what danger we incur from the possibility of septic fermentation. Besides, the author believes that the kneading of a tonically contracted uterus is, in itself, an injurious procedure. He therefore chooses the natural or expectant plan, interfering, however, by extraction of the after-birth as soon as loosened. Here, also, comes his innovation: Remembering that with or after every contraction the uterus rises toward the umbilicus, he feels for the fundus. If the placenta be undetached, it will have a round form; if it be loose, the fundus gives the impression of the margin of a cake, i. e., like a crest, (kammformig). At this moment he gently grasps the inferior segment directly above the symphysis, and pushing the whole organ toward the umbilicus, slowly, and without force, the after-birth drops out immediately. A

little traction on the cord is equally allowable. As far as time is concerned, the doctor thinks it ridiculous to specify it in minutes. He takes into consideration all the condition used, therefore, makes his conclusions as to active interference in cases too much prolonged. He believes in strict antisepsis.

In view of the frequency of the disfiguring cicatrices of burns about the hand, it might be interesting to hear of a new and comparatively successful proceeding both as to the final cosmetic effect and the absolute success of the operation. As is known, those cicatricial contractions are usually the result of burns. They may also follow extensive laceration of the hand by machinery, and another more rare etiological factor is gangrene. It must be added right here, that cases of gangrenous origin are rarely amenable to the treatment, since the process has usually caused extensive adhesions between the tendons and their sheaths. For the same reason the so-called contractions of Depuytren must be excluded. In all cases the little finger and thumb suffer most, the former on account of its position during falling, the latter on account of its need of almost unlimited mobility. Occasionally all fingers may be bound down into the palmar surface of the hand. In minor cases, where cicatricial bands are surrounded by healthy, movable skin, the adventitious band may be removed, and the loss repaired immediately by the V. and W. incision so well known in plastic surgery. If implicating the whole hand, palmar surface most usually, limit the palm by an incision skin-deep. Then dissect up carefully all the fibrous cicatricial tissue, looking out for vessels and nerves as much as possible, although they cannot always be avoided. If a tendinous sheath be cut it must be ligatured with catgut, antiseptic measures being observed throughout. This done, stretch the fingers while the hand is placed on a table. Having obtained a satisfactory position the second operation begins, i. e., the formation of a flap, before, it may be stated that the incision of the palmar wound be made so as to present an undermined edge, so as to fit the flap is the corresponding lateral wall of the chest, because the skin is extremely elastic and will cover eventual little insufficiency. Its vasenlarity is also great, and besides, the scar thus formed is not visible. The root of the flap ought to be toward the anterior aspect o the chest, and have a considerable width. The subcutaneous fat ought to be dissected up with the flap, at least around the margins and near the root. This will secure both an early union and prevent the drying up of the flap. The arm may easily be fixed so as to be entirely immovable, by a plaster of Paris dressing with a hole left over the extent of the wound. The operation is advisable only when some active movement of the fingers is manifest, showing that the sheaths are yet intact. To try to dissect up the cicatricial contractions *only*, and leave the healing to granulations would leave again a shrinking of the healing tissues as a consequence according to the law of general pathology.

Prof. Charcot, in speaking on the subject of Wieniere's disease, (vertigo) advised very urgently the employment of the salts of quinine. It is to be given in quantities of gr20-30 a day, and to be continued for long periods. In his consideration it is the most efficient drug for the malady. If space did not prevent it, a little description of his clinical lectures might be interesting not only on account of the great variety of affections, but particularly for the researches and experiments he makes on the subject of hypnotism. In fact, here it is usually called Charcotism. Not only students, but persons of all trades and stations assemble there, and the papers bring long reports. Of course, this is a little against our American ideas of propriety.

The diagnostic value of the crepitant rale in fibrous (croupous) pneumonia is formulated in the following theses, by Prof. Bernheim, of Nancy:

1. It may be present during the first period (engorgement), be absent during the second (hepatization), and reappear during the process of resolution.

2. Although during the third stage we usually encounter the subcrepitant rale, we may also meet the true variety.

3. The crepitant rale may be absent during the whole march of the disease, but put in its appearance during resolut,on.

4. Can exist in all stages.

5. Can be absent in all periods.

6. Can be replaced by the subcrepitant rale throughout.

7. [illegible]

subcrepitant in the two succeeding.

8. The preceding order (as given under 7) may be exactly reversed.

9. In the great majority the true crepitant rale is present in acute croupous pneumonia. When totally absent, hronchitis has supervened, the mucous rales of which, rendered sonorous by the solidity of the hepatized lung, cover the fine crepitant rale.

